# Current Techniques in Surgical Correction of Congenital Ptosis

**DOI:** 10.4103/0974-9233.63073

**Published:** 2010

**Authors:** Felicia D. Allard, Vikram D. Durairaj

**Affiliations:** Department of Ophthalmology, Division of Oculoplastic and Orbital Surgery, Rocky Mountain Lions Eye Institute, University of Colorado, Aurora, CO, USA

**Keywords:** Complications, Congenital Ptosis, Frontalis Sling, Levator Advancement, Mullerectomy, Ptosis Complications

## Abstract

Ptosis refers to vertical narrowing of the palpebral fissure secondary to drooping of the upper eyelid to a lower than normal position. Ptosis is considered congenital if present at birth or if it is diagnosed within the first year of life. Correction of congenital ptosis is one of the most difficult challenges ophthalmologists face. Multiple surgical procedures are available including, frontalis sling, levator advancement, Whitnall sling, frontalis muscle flap, and Mullerectomy. Selection of one technique over another depends on the consideration of several factors including the surgeon experience, the degree of ptosis in the patient, as well as the degree of levator muscle function. Current recommendations for the correction of congential ptosis vary based on clinical presentation. Advantages and disadvantages of each of these procedures are presented with recommendations to avoid complications.

## BACKGROUND

**Figure 1:**
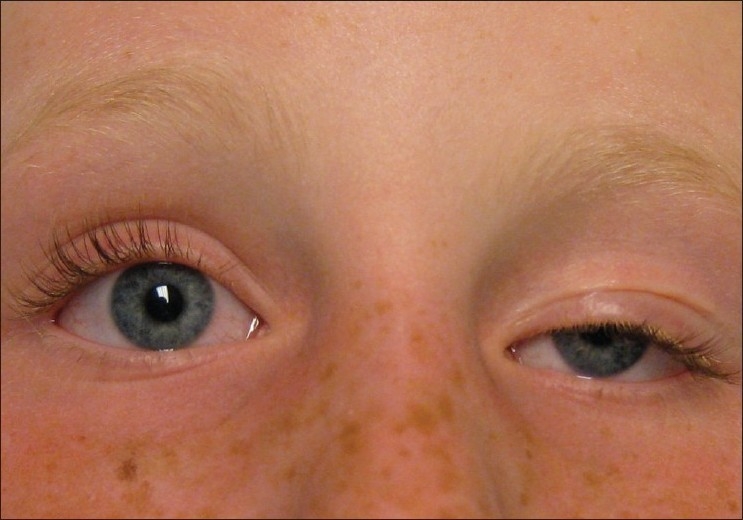
A patient with left upper eyelid congenital ptosis

Ptosis, an abbreviation for the term blepharoptosis, refers to vertical narrowing of the palpebral fissure secondary to drooping of the upper eyelid to a lower than normal position. Ptosis is considered congenital if present at birth or if it is diagnosed within the first year of life. Congenital ptosis is generally unilateral (70%), but may be bilateral, and can be isolated or associated with disease of one or more of the extraocular muscles and/or other systemic conditions.^1^,^2^ More severe forms may involve hypoplasia of the levator palpebrae superioris muscle or tendon with a minimal or absent eyelid crease.^3^ Children with this condition suffer from obstructed vision in their upper visual quadrants and frequently require surgery to elevate their eyelids [Figure 1].

Several genes that lead to isolated congenital ptosis or a syndrome involving congenital ptosis have been identified. These include PTOS1, PTOS2, and ZFH-4 which lead to autosomal-dominant forms of isolated congenital ptosis.^4^–^9^ Ptosis can also be seen as a component of many congenital syndromes including Duane retraction syndrome, blepharophimosis ptosis epicanthus inversus syndrome, congenital fibrosis of extraocular muscles, Marcus Gunn jaw winking syndrome, and congenital Horner's syndrome. Presently, very few studies have been conducted to track the prevalence of these genes or the prevalence of congenital ptosis. The only large-scale epidemiological data available is from the Section of Ophthalmic Genetics in China.^10^ Data from this registry reported a prevalence of congenital ptosis of 0.18% (1:552) in this population.^10^ This data was obtained from a registry containing information from China only and it is difficult to predict whether the incidence reflective of other populations.

Congenital ptosis is generally considered a nonprogressive condition; however, it is associated with the development of visual disturbances such as myopia, astigmatism, anisometropia, amblyopia, ocular torticollis, and strabismus. These sequelae of ptosis provide a compelling reason to pursue early surgical correction.^11^,^12^ This is particularly true in cases of congenital ptosis since clearance of visual axis is essential for prevention of visual loss related to amblyopia. After careful examination and establishment of the diagnosis of congenital ptosis, the need for possible surgical options should be carefully discussed with the parents of a child.

### Surgical considerations

The original surgical technique for the correction of ptosis utilized resection of upper lid skin to more effectively allow the frontalis muscle to elevate the eyelid. This method was found to be temporarily successful, failing after the skin stretched and eyelid returned to its original position. Failure of skin excision methods led to the modern, muscle-based surgical techniques. Maintenance of correct eyelid position is an important consideration when selecting a technique to correct congenital ptosis. Other considerations which have shaped the evolution of these surgical techniques include the need for cosmetically acceptable results, preservation of the normal eyelid crease, maintenance of the normal tear film, and prevention of exposure keratopathy by prevention of over correction.

## SURGICAL TECHNIQUES

### Traditional frontalis sling

**Figure 2:**
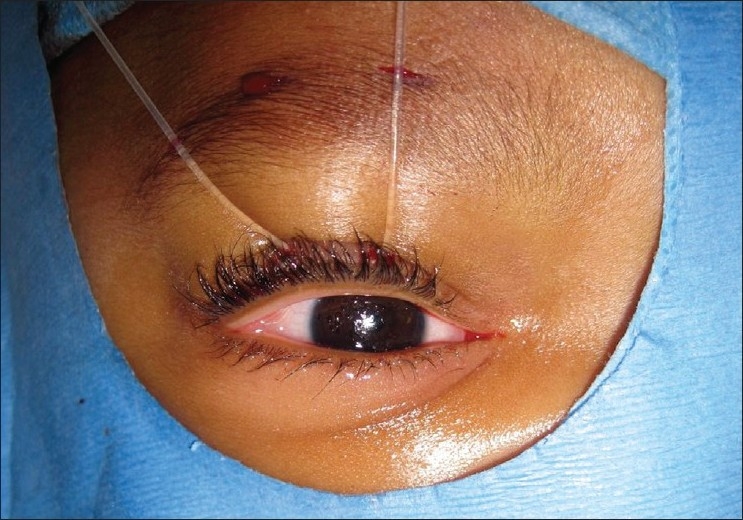
Intraoperative photo of a patient having frontalis sling suspension of eyelid

One surgical approach to patients with congenital ptosis and poor levator function or congenital Marcus Gunn jaw wink phenomenon is the traditional frontalis sling. This procedure involves creation of a linkage between the frontalis muscle and the tarsal and epitarsal tissue of the upper eyelid, which allows for a better eyelid position in primary gaze [Figure 2]. This allows eyelid elevation to be performed via the use of the frontalis muscle, thereby bypassing a poorly functioning levator.^13^ Disadvantages of this procedure include the risk of lagophthalmos and eyelid lag in down gaze.^14^ Other cosmetic considerations include scarring in young children, unsatisfactory geometric tenting of the pretarsal and preseptal skin, loss of the eyelid crease, and a poor tarso-corneal interface seen upon brow elevation and down-gaze. These cosmetic defects may be related to the choice of sling material and position of the material within the eyelid.^13^ The recurrence rate of ptosis after 20 months postoperatively is 26%.^13^

Currently, several types of materials are available to create the sling between the frontalis muscle and the eyelid tarsus. Some of these include autogenous or banked fascia lata and alloplastic materials that include chromic gut, collagen, polypropylene, silicone, stainless steel, silk, nylon monofilament, polyester, and polytetrafluoroethylene (PTFE).^13^ Fascia lata is not always a viable option in all patients as they need to be at least 3 years of age in order to have adequate leg length to provide suitable fascia lata.^15^ Ben Simon *et al*.^13^ reported that PTFE slings produced the lowest incidence of ptosis recurrence (15% compared to 30% from other materials), and that nylon slings produced the best cosmetic results.

Two sling placement patterns have been developed for the frontalis sling method: single loop or double pentagon sling techniques. No difference in recurrence, function, or cosmetic result has been reported between these two commonly performed techniques.^13^ Two incisional approaches have also been developed: the eyelid crease incision and the supralash stab incision. A study comparing the function and cosmetic results of these two incisional techniques found that the eyelid crease approach yields better results in terms of lid contour and lid crease symmetry.^16^

### Levator resection and advancement

**Figure 3:**
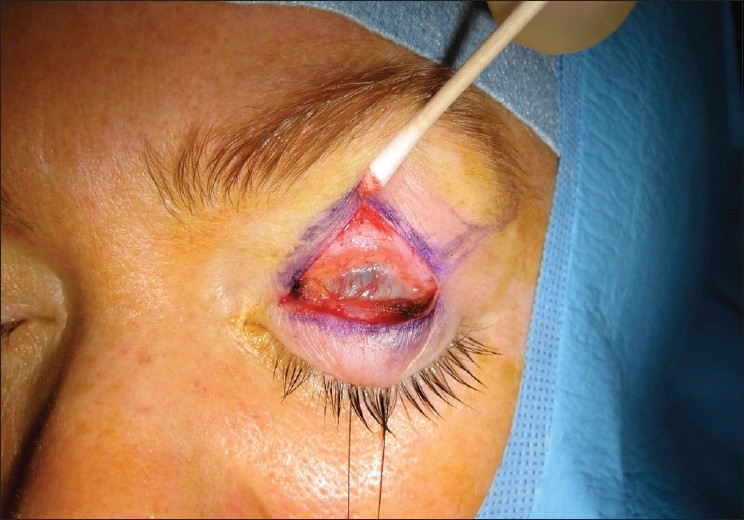
External levator approach to congenital ptosis repair

Resection and advancement of the levator aponeurosis is a technique often used in correction of ptosis in patients with greater than 5 mm of levator function. This technique is performed via the exposure of the levator aponeurosis through an anterior approach, traditionally using an incision running the entire length of the upper eyelid crease, then advancing the levator aponeurosis by folding or excising the muscle, and reattaching the aponeurosis to the anterior surface of the tarsus [Figure 3]. This method results in an elevation in the contour of the upper lid by effectively shortening the levator muscle itself.^17^ This approach has the advantages of preserving normal anatomical planes and structures of the eyelid as well as preservation of all elevating structures, including Mueller's muscle and Whitnall's ligament.^18^ This procedure may also be carried out using a trans-conjunctival approach or a small skin incision (8–13 mm) which requires minimal local anesthetic agent, causing less distortion of the tissue arguably leading to better cosmetic results and faster recovery.^19^ Disadvantages of the small incision approach are related to the limited view of the surgical field.

Cates and Tyers *et al*.^20^ reported their results from a series of 100 patients (108 eyelids) with congential ptosis and levator function of greater than 4 mm who underwent correction via anterior levator advancement. From their study, 76% of unilateral cases had a successful outcome at 6 weeks postoperatively (as measured by eyelid level being within 1 mm of each other side-to-side) falling to 74% at 6 months postoperatively.^20^ The most common postoperative side effects at 6 months were undercorrections in 19% of cases and overcorrections in 7% of cases.^20^ In this retrospective study, the authors found that the factor most predictive of outcome was levator function with increasing levator function leading to increasing risk of overcorrection.^20^

## WHITNALL SLING

The Whitnall sling procedure is used to correct severe ptosis with levator function of 3–5 mm. The Whitnall's ligament acts as a support structure for the upper eyelid and superior orbit by functioning as a superior suspensory ligament for the eyelid and lacrimal gland and a pulley off of which the levator muscle gains support and direction.^21^,^22^ The Whitnall sling procedure takes advantage of this second support structure of the eyelid. This procedure involves resecting the levator aponeurosis up to the point of Whitnall's ligament (maximal levator resection), and then suturing both Whitnall's ligament and the underlying levator muscle to the superior portion of the tarsal plate. If the suturing of Whitnall's ligament to the tarsus does not provide satisfactory lid elevation, a superior tarsectomy may be performed in addition to the sling procedure.^22^ This approach offers many of the same benefits as aponeurosis advancement: preservation of the levator muscle, Müller's muscle, and Whitnall's ligament without altering the structures that produce the three-layer tear film. One potential complication of the Whitnall sling is mistaking the lower-positioned transverse ligament (LPTL) for Whitnall's ligament. If the LPTL is mistakenly fixed to the tarsus, the elevation will be insufficient.^23^

Eyelid eversion may result if Whitnall's ligament is advanced too far down the tarsal plate. Other reported complications include corneal exposure (up to 100% of patients in the early postoperative period), overcorrection, undercorrection, poor eyelid contour or crease, lagophthalmos and conjunctival prolapse.^22^,^24^ Undercorrection was seen more often over time in the reported series.^22^,^24^ Anderson *et al*.^22^ reported a series of 69 ptotic eyelids that were corrected with Whitnall's sling without tarsectomy. Over a one-year follow-up, 20 out of 65 (30.7%) of the eyelids which were originally considered satisfactory (lid height within 2 mm of the contralateral lid) became unacceptable and required reoperation.^22^ This high incidence of late undercorrection seen led to the recommendation that the Whitnall's sling procedure be augmented with tarsectomy in most cases, especially ones with very poor levator function.^22^,^24^

## FRONTALIS MUSCLE FLAP

The frontalis muscle flap procedure is recommended for use in cases of severe ptosis with levator function less than 4 mm. Historically, this procedure evolved from the frontalis sling procedure. The flap procedure involves elevating the innervated frontalis muscle flap, passing it over a pulley created near the insertion of the orbital septum at the superior orbital rim, which redirects the pull of the frontalis to elevate the lid, and then attaching the frontalis muscle to the tarsal plate.^15^ The advantages of this technique include eliminating the need for alloplastic or autologous tissue to connect the caudal portion of the frontalis muscle to the eyelid as well as improving the direction of the pull.^15^ In this case, the pulley lifts the eyelid toward the brow rather than along the surface of the globe.^15^ Additional advantages over the frontalis sling procedure include minimal ptosis on upward gaze, less lid lag on downward gaze, preservation of eyelid contour and reduced tendency for the eyelid to pull away from the eye. This procedure may also be performed on patients at a younger age than a traditional frontalis sling because the frontalis muscle is well-developed by two years of age whereas fascia lata maturation may take an additional year.^25^

Complications of frontalis muscle flap include: (1) transient postoperative forehead anesthesia with spontaneous recovery, (2) eyebrow asymmetry, (3) reduced eyelid excursion with extreme upward and downward gaze, (4) lagophthalmos, and (5) overcorrection possibly due to the frontalis muscle being stronger than the levator muscle. In one report that followed 42 patients, no exposure keratopathy was reported; however, eyelid asymmetry was seen in 14% of patients, more frequently in unilateral cases than in bilateral cases.^15^ Similar results were reported in smaller studies.^25^,^26^ One study did report a single case of lagophthalmos complicated by corneal erosion.^27^

## MULLERECTOMY

Müller's muscle-conjunctival resection surgery is another technique that may be used to advance the levator aponeurosis of the upper eyelid to correct ptosis. The success of this procedure is dependent upon the combined function of the Muller's muscle and levator muscle hence use recommended in patients with fairly good muscle function.^28^ The Muller's muscle is an involuntary, sympathetically innervated muscle that originates below the levator aponeurosis just distal to the Whitnall's ligament. It attaches to the superior tarsal border by a small tendon and is responsible for an estimated 2–3 mm of eyelid elevation.^29^ This particular procedure is recommended for patients who respond well to the phenylephrine test, thereby shortening a responsive Muller's muscle.^29^ There are concerns, however, that the phenylepherine test may be inaccurate in predicting surgical results in over 20% of patients, especially those with Hering dependence.^30^ In this procedure, the conjunctiva and Muller's muscle are separated from the levator aponeurosis, the anterior aponeurosis is then bluntly dissected and separated from the orbicularis muscle. The aponeurosis and Muller's muscle are then sutured to the anterior aspect of the tarsal plate.^28^,^29^ Complications include corneal abrasions or ulcerations that are generally secondary to suture material irritation, hemorrhage, infection, and over- or undercorrection. In a report of 77 patients by Mercandette *et al.*, three suffered corneal abrasion, two required additional surgery (one for overcorrection and one for undercorrection).^29^ A criticism of this procedure is that unlike other techniques, such as the levator resection, the Mullerectomy does not allow for intraoperative adjustment of the eyelid height, rather an estimation of the proper resection length must be made preoperatively using various reported ratios of muscle resection to the eyelid elevation. Mercandetti *et al*. reported a ratio of 1.0 mm of resection for 0.32 mm of elevation.^29^

## DISCUSSION

In summary, correction of congenital ptosis in young children is arguably one of the most difficult challenges for ophthalmologists. Many authors have reported difficulty not only with adequately correcting ptosis, but also with functional and cosmetic results. There are currently several surgical options for the treatment of congenital ptosis. Selection of one technique over another will depend on consideration of several factors including the experience and comfort level of the surgeon with various techniques, the degree of ptosis in the patient, as well as the degree of levator muscle function. Current recommendations for the correction of congential ptosis vary by clinical scenario. In children less than 3–4 years of age in which levator function is poor, the frontalis sling procedure is recommended. In children with less than 3 mm of levator function, options include the frontalis sling, Whitnall sling (for very poor levator function),^22^ and dynamic frontalis muscle flap.^15^ In patients with more than 5 mm of levator function, levator resection and advancement may be used.^20^,^22^ For patients in which primary correction has failed, Whitnall sling procedure with or without tarsectomy is recommended after failed frontalis sling,^2^ and frontalis sling procedure is recommended for re-correction after failed Whitnall sling.^22^
